# Leadless Pacemaker Implantation in Severe Kyphosis: Challenging Implantation for Tortuous IVC and Narrow RA

**DOI:** 10.1016/j.jaccas.2024.102295

**Published:** 2024-03-18

**Authors:** Shogo Sakamoto, Tomomi Tani, Kenji Baba, Shiho Wakasa, Moritoshi Irishio, Toru Kataoka, Daiju Fukuda

**Affiliations:** a Department of Cardiovascular Medicine, Belland General Hospital, Higashiyama, Naka-ku, Sakai, Osaka, Japan; b Department of Cardiovascular Medicine, Osaka Metropolitan University Graduate School of Medicine, Asahimachi, Abeno-ku, Osaka, Japan

**Keywords:** computed tomography, kyphosis, leadless pacemaker

## Abstract

Leadless pacemaker implantation is recognized as safe and effective for treating bradycardia. However, there are limited descriptions of its use in patients with complex anatomical considerations. Here, we present a case detailing the successful implantation of a leadless pacemaker with a tortuous inferior vena cava and a narrow right atrium.

## Graphical abstract

**Figure:**
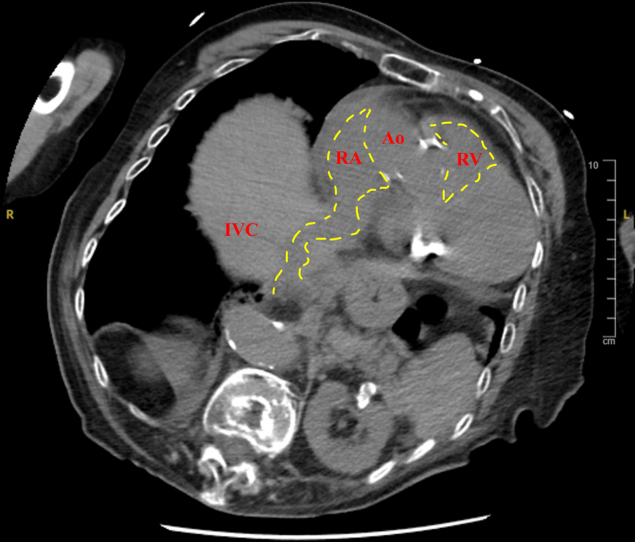

## History of Presentation

**Figure 1:**
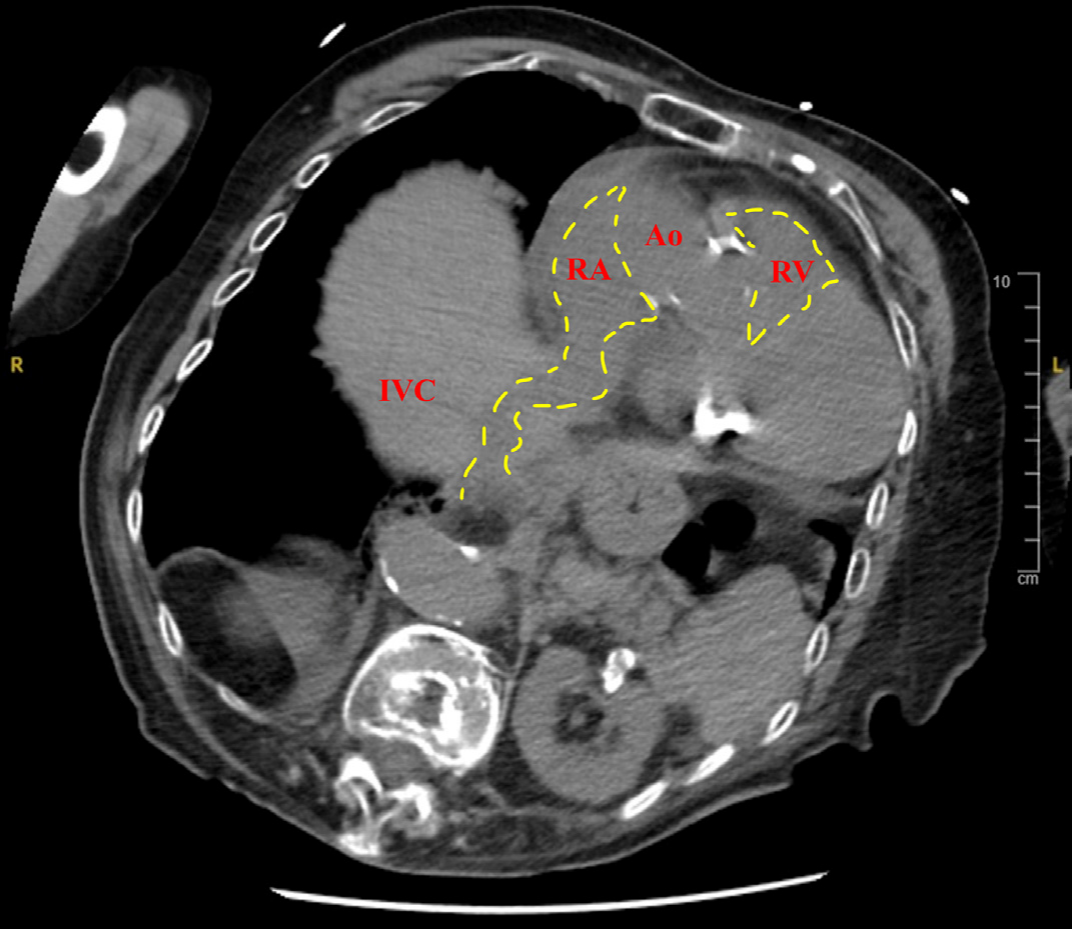
Computed Tomography Computed tomography image showing anterior elongation of the inferior vena cava (IVC)–right atrium (RA) junction (yellow dashed lines) caused by the kyphosis. Ao = aorta; RV = right ventricle.

**Figure 2:**
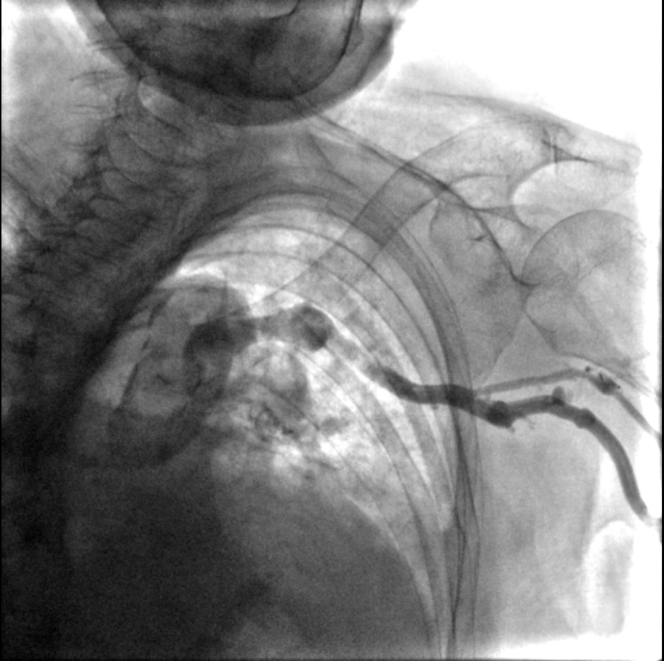
Left Subclavian Venography Venography shows that the left subclavian vein drains into the superior vena cava with bending.

A 91-year-old emaciated woman (height 147 cm, weight 43 kg, body mass index 19.9 kg/m^2^) was urgently hospitalized for heart failure. Electrocardiography revealed a complete atrioventricular block. Chest radiography indicated congestive heart failure and kyphosis. Computed tomography (CT) indicated a tortuous inferior vena cava (IVC) and narrow right atrium (RA) (Figure 1). Left subclavian angiography revealed a venous tortuous route to the RA (Figure 2). The patient was cognitively impaired, and her activity of daily living was expected to decline with prolonged hospital period. Additionally, because of the severe kyphosis and thin thoracic skin (subcutaneous thickness 3.2 mm), we decided to forego the implantation of a conventional transvenous pacemaker and insert a leadless pacemaker (LP).

**Learning Objectives**

• To perform volume-rendered CT is useful to confirm the tortuous IVC that drains into the RA in patients with severe kyphosis.
• To repeat venography and confirm the route to the RA is necessary before implanting LP in a patient with a severely tortuous IVC.

## Past Medical History

The patient was diagnosed with hypertension and had been taking calcium antagonists.

## Differential Diagnosis

A possible cause of IVC dilation and tortuosity is chronic pulmonary hypertension. However, in this case, the absence of a history of lung disorder, coupled with the meandering of both IVC and aorta, led us to consider kyphosis as the likely cause.

## Investigations

LP implantation commenced under awake conditions because of concerns about the potential loss of junctional rhythm during sedation. We attempted to insert a 5.0-F pigtail catheter through a 9.0-F introducer sheath via the right femoral vein and advance it into the right ventricle (RV) to enable right ventriculography. Subsequently, attempts to advance the pigtail catheter into the superior vena cava were unsuccessful (Video 1). Venography from the IVC revealed a narrow RA and severe tortuosity in the IVC draining into it (Video 2).

## Management

**Figure 3:**
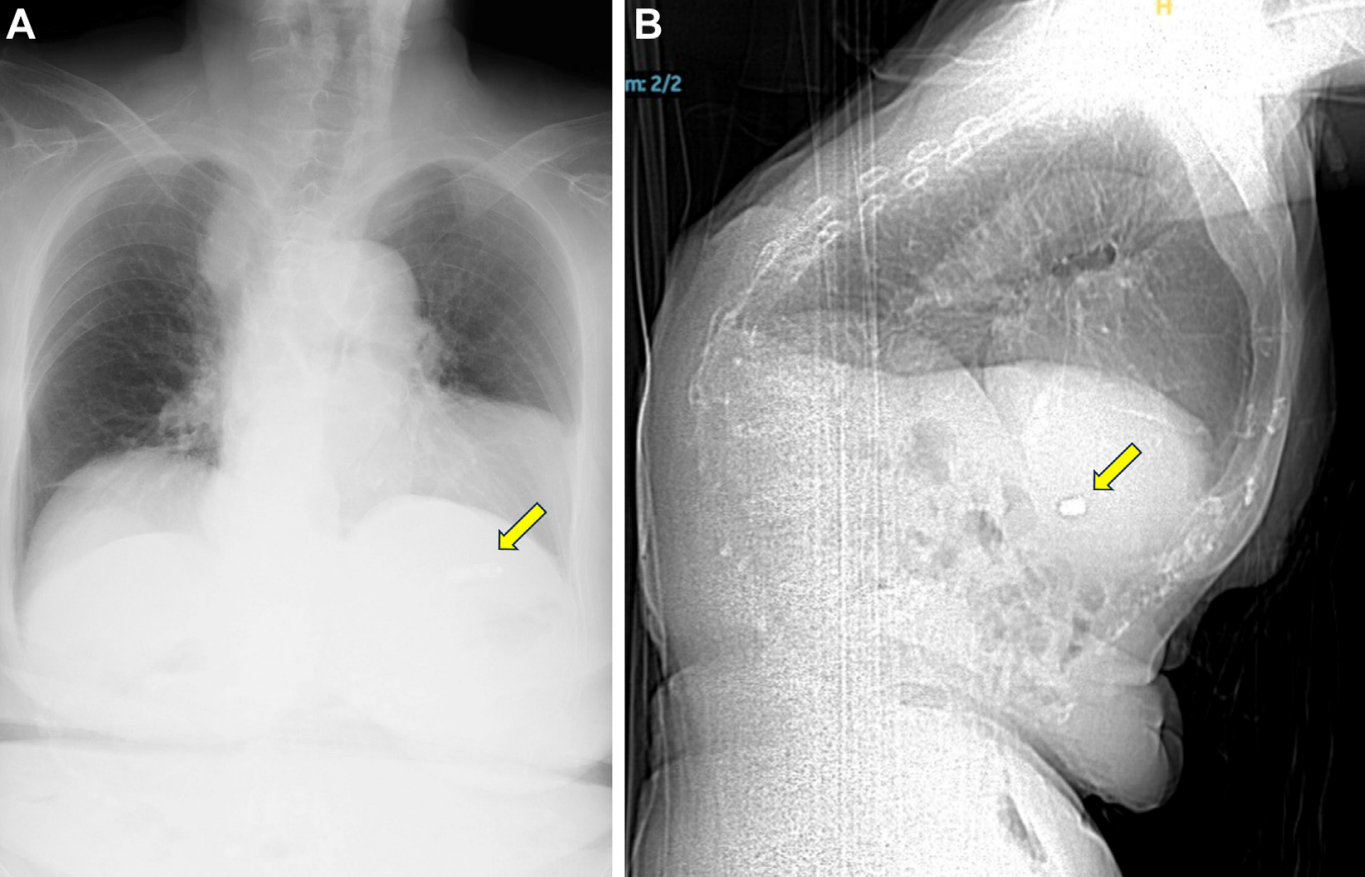
Radiography and Computed Tomography (A) Chest radiography and (B) sagittal-view computed tomography image showing anterior displacement of the heart caused by the barrel chest deformity and leadless pacemaker placement (yellow arrows).

We replaced the 9.0-F introducer sheath with a 27-F hydrophilic sheath to facilitate the gradual advancement of a 23-F steerable delivery catheter for the LP into the IVC. In case of resistance, we retracted the sheath and repeated the process, performing venography for guidance (Video 3). After successfully navigating the delivery sheath across the summit of the bent IVC, it was smoothly manipulated across the tricuspid valve into the RV. However, there was some difficulty with the steerable delivery catheter reaching the RV septum because of the anterior displacement of the heart relative to the thoracic deformity. We advanced the delivery catheter toward the free wall of the RV to create an alpha loop and successfully directed the LP to the RV side of the septum (Video 4). With advancing the delivery catheter further, LP was successfully guided into the RV side of the apical septum. We used contrast angiography to confirm contact between the LP and the trabeculae of the RV septum (Video 5). Following confirmation, the LP was deployed, and the tether wire was cut caused by the satisfactory performance of the LP on the initial deployment. The pacing threshold at the implant site was 1.0 V at 0.24 ms, sensing was 10.5 mV, and pacing impedance measured 660 Ω. Finally, a suture-mediated device was employed for the closure of the femoral access site. There were no procedural complications, and chest radiography and volume-rendered CT confirmed the LP’s location and the right heart’s anatomy (Figures 3A and 3B, Video 6).

## Discussion

LP implantations are generally safe and effective with standard access routes.^1^ However, preimplantation risk assessment is important. Piccini et al^2^ reported a risk score for predicting pericardial effusion in patients undergoing LP implantation, and the number of deploys was linked to effusion in each risk group. Our patient, with a risk score of 4, was deemed high risk, necessitating the need for a successful LP implantation in the first attempt. Despite the conventional pacemaker implantation and LP implantation being anatomically challenging procedures in our case, LP implantation was chosen for early discharge. Fortunately, a successful LP implantation was achieved on the first attempt.

There have been several reports related to anatomical complex patients.^3^, ^4^, ^5^ A preoperative CT scan can reveal various anatomic abnormalities and is useful for LP implantation planning. However, there are limited reports of cases involving kyphosis. Arana-Rueda et al^6^ reported a case of LP implantation in a patient with kyphosis. Fortunately, the authors were able to successfully deliver the steerable sheath without any incidence using RA access. Kyphosis, which is commonly experienced in elderly patients, causes RA and RV dilatation and anterior elongation of the IVC–RA junction. However, venous tortuosity degree and LP implantation difficulty depend on kyphosis severity.

In our case, delivering the steerable sheath to the RA proved challenging caused by severe IVC tortuosity. Additionally, the abnormal position of the RV prevented the advancement of the LP to the right septum.

We believe that repeat venography is useful for navigating a tortuous venous system with the steerable delivery sheath. Additionally, in cases where the right chamber rotates backward, advancing the LP into the RV septum may be facilitated using an alpha-looped delivery sheath in the RV.

## Follow-up

We observed no issues during the final postimplantation device interrogation at 7 days postimplantation. The patient was discharged 9 days later without heart failure or any other complications.

## Conclusions

Severe kyphosis alters venous routes and causes cardiac structural changes. Currently, there is no established technique for LP implantation in patients with a severely tortuous IVC. Therefore, employing volume-rendered CT is essential to understand structural changes in veins and the heart before attempting LP implantation in patients with kyphosis. We believe that repeat contrast procedures to confirm the venous route can be useful for safe LP delivery to the RV.

## Funding Support and Author Disclosures

The authors have reported that they have no relationships relevant to the contents of this paper to disclose.

## Appendix

Video 1 The Pigtail Catheter Could Not Be Advanced Through the Superior Vena Cava and Was Led Smoothly to the Apex of the Right Ventricle

Video 2 Venography From the IVC Shows the Tortuous IVC and the Narrow Right Atrium

Video 3 The Contrast From the Delivery Catheter Helped Confirm the Current Location and Advancement of the Leadless Pacemaker

Video 4 The Process of Creating the Alpha-Loop Was to Advance the Delivery Sheath to the Free Wall of the RV and Apply Deflection

Video 5 Creating the Alpha Loop With the Steerable Sheath Allowed the Leadless Pacemaker to Reach the Right Ventricle Septum Vertically

Video 6 Volume-Rendered Computed Tomography Scan Showing an Overview of the Bent Inferior Vena Cava and the Deformed Right Atrium
